# Supplementary material for: Systematic comparison and reconstruction of sea urchin (Echinoidea) internal anatomy: a novel approach using magnetic resonance imaging
Source: BMC Biol. 2008 Jul 23;6:33. doi: 10.1186/1741-7007-6-33 (PMC2500006; doi:10.1186/1741-7007-6-33)
Supplement: Additional file 1 — Table 1 – Scanning parameters for specimens used in this study. [file 1741-7007-6-33-S1.pdf]

Table 1 - Scanning parameters for specimens used in this study.

Specimen	Scanning location	Scanning solution	Spatial resolution	Field of view (FOV)	Matrix size in pixel	Echo time (T_E)	Repetition time (T_R)	Average number	Scanning time	Figure in this study
Eucidaris metularia (NHM)	Berlin (7 T)	H_20 dest. + Magnevist	(81 µm)^3	(3.12 cm)^3	(384)^3	6.7 ms	30 ms	12	15 h	2A, 3, 6A-C, 3D model
Caenopedina mirabilis (USNM)	Berlin (7 T)	H_20 dest. + Magnevist	(81 µm)^3	(3.12 cm)^3	(384)^3	6.7 ms	30 ms	12	15 h	3
Diadema savignyi (Aquarium store)	Würzburg (17.6 T)	Formalin + Magnevist	(40 µm)^3	1.7x1.7x1.4 cm^3	420x420x348	2.84 ms	20 ms	22	18 h	3
Salenocidaris (= Salenia) hastigera (ZMB)	Berlin (7 T)	H_20 dest. + Magnevist	(81 µm)^3	(3.12 cm)^3	(384)^3	6.7 ms	30 ms	12	15 h	3
Arbacia lixula (= var. *africana)* (NHM)	Würzburg (17.6 T)	H_20 dest. + Magnevist	(44 µm)^3	1.7x1.5x1.7 cm^3	384x340x384	2.57 ms	20 ms	36	26 h	3
Stomopneustes variolaris (USNM)	Berlin (7 T)	H_20 dest. + Magnevist	(81 µm)^3	(3.12 cm)^3	(384)^3	6.7 ms	30 ms	12	15 h	3
Psammechinus miliaris (BAH)	Berlin (7 T)	H_20 dest. + Magnevist	(81 µm)^3	(3.12 cm)^3	(384)^3	6.7 ms	30 ms	12	15 h	1A-E
	Würzburg (17.6 T)	Formalin + Magnevist	(44 µm)^3	1.7x1.4x1.7 cm^3	384x320x384	2.97 ms	20 ms	22	15 h	2B
Psammechinus miliaris (ZMB)	Berlin (7 T)	H_20 dest. + Magnevist	(81 µm)^3	(3.12 cm)^3	(384)^3	6.7 ms	30 ms	12	15 h	1F
Strongylocentrotus purpuratus (CAS)	Berlin (7 T)	H_20 dest. + Magnevist	(81 µm)^3	(3.12 cm)^3	(384)^3	6.7 ms	30 ms	12	15 h	3
Mespilia globulus (ZMB)	Würzburg (17.6 T)	H_20 dest. + Magnevist	(36 µm)^3	(1.4 cm)^3	(384)^3	2.65 ms	20 ms	40	33 h	3
Echinoneus cyclostomus (NHM)	Berlin (7 T)	H_20 dest. + Magnevist	(86 µm)^3	(3.12 cm)^3	(384)^3	6.7 ms	30 ms	12	15 h	2C, 4, 5A
Echinolampas depressa (USNM)	Berlin (7 T)	H_20 dest. + Magnevist	(81 µm)^3	(3.12 cm)^3	(384)^3	6.7 ms	30 ms	12	15 h	4, 5B
Echinocyamus pusillus (BAH)	Würzburg (17.6 T)	Formalin + Magnevist	20x18x18 µm^3	5.0x4.5x4.5mm^3	(256)^3	3.27 ms	20 ms	32	12 h	2D, 4
Pourtalesia wandeli (NHM)	Berlin (7 T)	H_20 dest. + Magnevist®	(86 µm)^3	(3.3 cm)^3	(384)^3	6.7 ms	30 ms	12	15 h	4, 5C
Abatus (= Hemiaster) cavernosus (ZMB)	Berlin (7 T)	H_20 dest. + Magnevist	(81 µm)^3	(3.12 cm)^3	(384)^3	6.7 ms	30 ms	12	15 h	4, 5D
