# Supplementary material for: Systematic comparison and reconstruction of sea urchin (Echinoidea) internal anatomy: a novel approach using magnetic resonance imaging
Source: BMC Biol. 2008 Jul 23;6:33. doi: 10.1186/1741-7007-6-33 (PMC2500006; doi:10.1186/1741-7007-6-33)
Supplement: Additional file 2 — Table 2 – List of specimens used in this study. [file 1741-7007-6-33-S2.pdf]

Table 2 - List of specimens used in this study.

Specimen	Specimen ID	Source	Locality	Year collected	Collected by	Identified by	Diameter without spines	Figure in this study
Eucidaris metularia (Lamarck, 1816)	1969.5.1.15-40	NHM	Aldabra, Indian Ocean	1969	J.D. Taylor	Unknown	2.0 cm	2A, 3, 6A-C, 3D model
Caenopedina mirabilis (Döderlein, 1885)	31182	USNM	Kii Strait, Pacific Ocean	1906	Northwestern Pacific Expedition	H.L. Clark	1.5 cm	3
Diadema savignyi Michelin, 1845	n/a, one specimen	Aquarium store	Red Sea, Indian Ocean	2006	Seewasser Center Berlin	A. Ziegler	1.9 cm	3
Salenocidaris (= Salenia) hastigera (Agassiz, 1869)	5816	ZMB	Maldives, Indian Ocean	1898	Valdivia Expedition	Unknown	1.5 cm	3
Arbacia lixula (= var. *africana*) (Linnaeus, 1758)	1952.3.26.31-36	NHM	Ghana, Atlantic Ocean	1949	R. Bassindale	Unknown	1.8 cm	3
Stomopneustes variolaris (Lamarck, 1816)	E45930	USNM	Greater Sunda Islands, Indian Ocean	1963	L. Kelts	C. Ahearn	2.2 cm	3
Psammechinus miliaris (Müller, 1771)	n/a, several specimens	BAH	Helgoland, North Sea	2006	BAH	BAH	1.5-2.5 cm	1A-E, 2B
Psammechinus miliaris (Müller, 1771)	2011	ZMB	Arendal, Baltic Sea	1872	Pommerania Expedition	Unknown	1.7 cm	1F
Strongylocentrotus purpuratus (Stimpson, 1857)	5724	CAS	Morro Bay, Pacific Ocean	1972	J.T. Carlton et al.	J.T. Carlton et al.	2.3 cm	3
Mespilia globulus (Linnaeus, 1758)	5620	ZMB	Madang, Pacific Ocean	1909	S. Schöde	Unknown	1.3 cm	3
Echinoneus cyclostomus Leske, 1778	1969.5.1.105	NHM	Aldabra, Indian Ocean	1969	J.D. Taylor	Unknown	Length: 3.4 cm	2C, 4, 5A
Echinolampas depressa Gray, 1851	E32955	USNM	Florida, Gulf of Mexico	1981	Continental Shelf Associates	Continental Shelf Associates	Length: 2.8 cm	4, 5B
Echinocyamus pusillus (Müller, 1776)	n/a, one specimen	BAH	Helgoland, North Sea	2006	BAH	BAH	Length: 5.0 mm	2D, 4
Pourtalesia wandeli Mortensen, 1905	1976.7.30.76-95	NHM	300 mls S of Iceland, Atlantic Ocean	1976	Discovery Expedition	Unknown	Length: 3.3 cm	4, 5C
Abatus (= Hemiaster) cavernosus (Philippi, 1845)	5854	ZMB	Kergueles, Indian Ocean	1898	Valdivia Expedition	Unknown	Length: 2.8 cm	4, 5D
