# Supplementary material for: Systematic comparison and reconstruction of sea urchin (Echinoidea) internal anatomy: a novel approach using magnetic resonance imaging
Source: BMC Biol. 2008 Jul 23;6:33. doi: 10.1186/1741-7007-6-33 (PMC2500006; doi:10.1186/1741-7007-6-33)
Supplement: Additional file 7 — 3D model – 3D PDF version of the entire article. By clicking anywhere onto Figure 6 the 3D model of Eucidaris metularia (Lamarck, 1816) can be interactively accessed. The 3D model is based on the virtual MRI sections seen in Video 1. In order to view the 3D model please install (or upgrade to) Adobe Reader Version 7.1 or higher. [file 1741-7007-6-33-S7.pdf]

Systematic comparison and reconstruction of sea urchin (Echinoidea) internal anatomy: a novel approach using magnetic resonance imaging

Alexander Ziegler*[1], Cornelius Faber[2,3], Susanne Mueller[4] and Thomas Bartolomaeus[1]

[1]Institut für Biologie, Freie Universität Berlin, Königin-Luise-Straße, 14195 Berlin, Germany.

[2]Experimentelle Physik 5, Universität Würzburg, Am Hubland, 97074 Würzburg, Germany.

[3]Institut für Klinische Radiologie, Universitätsklinikum Münster, Waldeyerstraße, 48149 Münster, Germany.

[4]Berlin NeuroImaging Center, Charité-Universitätsmedizin Berlin, Charitéplatz, 10117 Berlin, Germany.

Email: AZ*: aziegler@zoosyst-berlin.de

CF: faberc@uni-muenster.de

SM: susanne.mueller1@charite.de

TB: tbartol@zoosyst-berlin.de

*Corresponding author.

Abstract

Background: Traditional comparative morphological analyses and subsequent three-dimensional reconstructions suffer from a number of drawbacks. This is particularly evident in the case of soft tissue studies that are technically demanding, time-consuming, and often prone to produce artefacts. These problems can partly be overcome by employing non-invasive, destruction-free imaging techniques, in particular micro-computed tomography or magnetic resonance imaging.

Results: Here, we employed high-field magnetic resonance imaging techniques to gather numerous data from members of a major marine invertebrate taxon, the sea urchins (Echinoidea). For this model study, 13 of the 14 currently recognized high-ranking subtaxa (orders) of this group of animals were analyzed. Based on the acquired datasets, interactive three-dimensional models were assembled. Our analyses reveal that selected soft tissue characters can even be used for phylogenetic inferences in sea urchins, as exemplified by differences in the size and shape of the gastric caecum found in the Irregularia.

Conclusions: The main focus of our investigation was to explore the possibility to systematically visualize the internal anatomy of echinoids obtained from various museum collections. We show that, in contrast to classical preparative procedures, magnetic resonance imaging can give rapid, destruction-free access to morphological data from numerous specimens, thus extending the range of techniques available for comparative studies of invertebrate morphology.

Background

For centuries, comparative zoomorphological analyses have formed the backbone of phylogenetic inferences. Data on the external and internal morphology of specimens were obtained by dissecting and sectioning selected representative species. These traditional procedures are technically demanding as well as time-consuming and irretrievably alter or even destroy the specimen. Further complications arise from the difficult three-dimensional (3D) reconstruction of the structures observed. Artefacts, for example deformation or compression of histological slices, and the problematic alignment of sections make 3D reconstructions a laborious and often highly subjective procedure. The problems encountered are further aggravated by the logistic challenges that arise when comparative studies on selected species of a whole taxon are to be carried out. For example, in case of sea urchins (Echinoidea, Echinodermata), some species are deep-sea dwellers or confined to remote regions of the world. It is therefore a considerable problem to obtain these animals in a freshly fixed state. Understandably, the desirable use of specimens from museum collections is usually not permitted for invasive and destructive analyses.

The application of non-invasive imaging techniques such as micro-computed tomography (µCT; see, for example, [1-8]) or magnetic resonance imaging (MRI; see below) has substantially increased in basic morphological research. Whereas µCT is mainly used to display hard tissue such as bone or calcite structures, MRI provides images depicting primarily soft tissue anatomy. MRI is based on the principles of nuclear magnetic resonance (NMR) and is predominantly used to measure the distribution of hydrogen protons within a sample. Image contrast is achieved due to the different physical properties of tissues. Both µCT and MRI produce standardized digital data, principally permitting an interactive access through virtual collections or voxel libraries as envisaged previously [9, 10]. This data

represents the unaltered structural composition of the specimen, in contrast to classical histological or dissection techniques and apart from fixation. In the light of these and other recent technological advances, Budd and Olsson [11] refer to the renaissance of morphology, calling for a more extensive use of non-invasive and non-destructive imaging techniques and 3D applications. Since many of the specimens in natural history collections are conserved in alcohol, forming the so-called wet collections, non-invasive imaging techniques might be employed to reveal the internal and external anatomy of preserved, scientifically irreplaceable specimens. This extended use of museum specimens for comparative anatomical studies would allow for a substantial increase in taxon sampling on one hand, and on the other would minimize the ecological impact of such analyses.

The feasibility of MRI studies to reveal soft tissue characteristics of invertebrates was demonstrated by studies on crabs [12, 13], squid [14], crayfish [15, 16], oysters [17, 18], spiders [19], demosponges [20], and insects [21-29] (see also the review by Hart et al. [30]). Ziegler and Angenstein [31] used living and freshly fixed specimens of the sea urchin *Psammechinus miliaris* (Müller, 1771) and showed that members of this taxon are likely to be suitable for detailed MRI studies. These studies yielded imaging results comparable to manual dissection and were correlated with histological data. To achieve better resolved images of the complex internal anatomy of sea urchins, the application of higher magnetic field strengths was proposed [32]. However, all of the studies mentioned above were restricted to the analysis of single specimens or species in a non-systematic context.

For systematic analyses, however, sea urchins appear particularly suitable. Like sea stars or sea cucumbers, they are part of a major deuterostome taxon, the Echinodermata, which shares a common ancestor with diverse groups such as acorn worms (Enteropneusta), tunicates (Tunicata), and vertebrates (Vertebrata) [33]. Owing to the calcified skeleton found in sea

urchins, palaeontologists have access to a rich fossil record permitting thorough ground-truthing of phylogenetic hypotheses that are based upon molecular or morphological datasets [34, 35]. Recently, the importance of these animals for evolutionary and developmental inferences has been further emphasized by results obtained through the sequencing of the genome of the purple sea urchin, *Strongylocentrotus purpuratus* (Stimpson, 1857) [36].

Sea urchins are spherical, oval or flattened free-moving echinoderms covered with spines. Their soft tissue anatomy is characterized by a dominant digestive tract, a varying number of gonads, and the highly specialized water vascular system with its external appendages, the tube or ambulacral feet. The space between the major organ systems is composed of the main body cavities (the so-called perivisceral coeloms) that are filled with coelomic fluid [37]. Therefore, echinoids possess several of the internal structures encountered in most other taxa, although a number of highly specialized organs are present as well. This taxon can thus serve as a model for analyses using non-invasive imaging techniques on a broad systematic scale.

The purpose of our study was to explore the possibility of generating high-resolution 3D MRI datasets from selected specimens of a major invertebrate taxon. For the first time, a non-invasive imaging technique is employed for systematic comparative analyses. We show that MRI permits the rapid acquisition of digitized morphological data on a broad scale and the construction of 3D models that can be made interactively accessible on the Web. In addition, our approach can be employed to compare the internal anatomy of sea urchins to evaluate such datasets for evolutionary inferences.

Results

Establishment of imaging conditions

Initially, freshly fixed as well as museum specimens of *Psammechinus miliaris* (Müller, 1771), a common species in the North Sea, were used in order to establish imaging and contrasting protocols for broader systematic analyses. Living specimens of *P. miliaris* were made available through the Biologische Anstalt Helgoland, Germany (BAH). Several dozen sea urchins were kept in the aquarium facilities of the Institut für Biologie, Freie Universität Berlin, Germany, for subsequent scanning and contrasting experiments.

The first set of analyses was conducted to compare different specimens of freshly fixed sea urchins. Imaging was performed initially at a resolution of $(81\ \mu m)^3$ using different imaging protocols with specimens of almost identical size. This analysis showed that although slight differences in anatomical makeup did occur, localization, arrangement and overall shape of the internal organs did not differ significantly. In the specimens depicted (Figure 1A and B), this is exemplified by the almost identical shapes of both Aristotle's lanterns and the localization and shape of the digestive tract components. The structure of the wall of the sea urchin lower gut loop (also termed the stomach) was undulated in both specimens, whereas the horizontal sections of the upper gut loop (also termed the intestine) showed a smooth structure. The digestive tract of one specimen was partly filled (Figure 1A), while the digestive tract of the other specimen was empty, apart from tiny objects (Figure 1B). Another striking similarity was the almost exactly identical location of the inner and outer marginal sinus of the haemal system in both specimens. However, some differences were discernable, for example the distinct shapes of the ampullae and the thickness of the interpyramidal muscles, indicating that, ideally, several specimens of a given species should be scanned. Similar comparative experiments were performed on a total of 18 echinoid species (data not shown). In all cases, the overall internal architecture of two specimens of a given species resembled each other more closely than that of other species.

The next set of experiments served to assess the effects of a gadolinium-based contrast agent (Magnevist, a standard in clinical and pre-clinical MRI studies) on image quality. Using the same specimen of *P. miliaris* and the same imaging protocol, we found that the application of the contrast agent resulted in enhanced contrast, although some thin-walled structures, such as the ampullae or the oesophagus, became less visible (Figure 1C and D). Another advantage of the use of this contrast agent was that some of the artefacts caused by paramagnetic gut content were reduced due to the stronger water signal. Therefore, most specimens were scanned both before and after the application of the contrast agent (see Additional file 1 for details).

The final set of experiments for establishing a standard set of imaging conditions was designed to compare imaging results obtained from freshly fixed and museum specimens of the same species. Prior to the present MRI studies, several museum specimens from the wet collection of the Systematische Zoologie am Museum für Naturkunde, Berlin, Germany (ZMB) were dissected in order to assess the state of preservation after decades or even more than a century of alcohol conservation (*Strongylocentrotus dröbachiensis* (Müller, 1776), *Sphaerechinus granularis* (Lamarck, 1816), *Schizaster lacunosus* (Linnaeus, 1758), and *Brissopsis lyrifera* (Forbes, 1841)). Their state of preservation was surprisingly good and seemed mainly to depend on the mode of collection, the fixation mode, and the subsequent preservation in alcohol. Following the dissections, MRI experiments using freshly fixed and museum specimens of *P. miliaris* were carried out. Structures visible in the freshly fixed specimen (Figure 1E) were recognizable also in the 135-year-old museum specimen (Figure 1F). The size and location of the anatomical features varied slightly, but this had been observed among different specimens of freshly fixed animals as well. Despite these differences, the results confirmed our assumption that museum material, even if more than a hundred years old, can be used for systematic MRI studies.

Scanning of selected members of high-ranking echinoid subtaxa

After an initial assessment of the suitability (that is, size, state of preservation, and availability) of specimens in natural history collections worldwide, MRI studies were carried out using selected species of the 14 currently recognized echinoid subtaxa (orders). In three cases, freshly fixed animals were employed for the analysis (see Additional file 2 for details).

The full datasets from two ‘regular’ sea urchins (*Eucidaris metularia* (Lamarck, 1816) and *P. miliaris*) as well as two irregular sea urchins (*Echinoneus cyclostomus* (Leske, 1778) and *Echinocyamus pusillus* (Müller, 1776)) are presented here as interactive videos (Additional files 3-6; Figure 2 shows images of selected sections). The four image datasets range in spatial resolution from $20 \times 18 \times 18\ \mu m^3$ to $(86\ \mu m)^3$ and represent the current state-of-the-art in high-field MRI at a field of view of up to $(3.3\ cm)^3$. In *E. metularia* (Figure 2A) and *P. miliaris* (Figure 2B), the pentameric symmetry of echinoderms becomes easily visible when looking at the five gonads or the lantern muscles that form a five-tipped star. Moving down from the aboral to the oral side, the following internal and external structures can be identified, amongst others (Additional files 3-6): madreporic plate, gonads, spines, intestine, compass elevator muscles, Aristotle’s lantern with corresponding muscles, and the stomach. Some parts of the gut of *P. miliaris* were filled with paramagnetic sediment of unknown composition (possibly iron or manganese residues) that caused artefacts by altering the homogeneity of the magnetic field inside the MRI instrument.

In *E. cyclostomus* (Figure 2C) and *E. pusillus* (Figure 2D), the secondarily developed bilateral symmetry found in the Irregularia is obvious when looking at the gonads (reduced to four). The digestive tract of *E. cyclostomus* is characterized by a dominant gastric caecum, localized at the beginning of the stomach, and a long rectum, leading down to the anus which is situated

at the oral side of the animal. *E. pusillus*, in common with all members of the Clypeasteroida, the so-called sand dollars, possesses a modified Aristotle's lantern. Other structures that can be identified in the irregular species include relatively small spines, one dominant (*E. pusillus*) or two huge (*E. cyclostomus*) gut loops, filled with debris and detritus, and an axial complex running straight down from the madreporic plate to the adoral part of the oesophagus (*E. cyclostomus*). In addition to soft tissues, the delineation of calcareous structures caused by the signal-providing surrounding fluids and tissues adds some information on hard-part anatomy to the virtual sections as well.

Although several specimens of the echinoid taxon missing in our study, the Echinothurioida (or leather urchins), were found in museum collections, their shape prevented successful soft tissue imaging. These sea urchins possess a flexible calcite endoskeleton as well as specialized internal muscles that contract when the animal is fixed, resulting in a pancake-like shape of the organism. For a reliable comparison of internal structures it would be necessary to scan specimens prior to contraction of these powerful muscles.

3D reconstruction of selected internal organs

The 3D reconstructions presented here are limited to the major soft tissue structures identifiable in all datasets, if present in the respective species (Figure 3C-E and 4C-E): digestive tract, Stewart's organs, axial complex, siphon, gonads, buccal sacs (or 'gills') and gastric caecum.

Two of the immediately recognizable features are size and shape of the gonads. Both differ substantially between the analyzed species and usually constitute a character set of high intraspecific variability. This becomes evident from the analysis of animals from a given species in distinct developmental stages (data not shown). The specimen of *Stomopneustes*

variolaris (Lamarck, 1816) (Figure 3) shown here was fixed before gonad maturation. In the case of the highly branched gonads found in *E. pusillus* (Figure 4), depicted here for the first time, the advantages offered by the digitized reconstruction of morphological structures become obvious: a 3D reconstruction of such complex structures would be particularly demanding using traditional techniques. In contrast to the gonads, clearly distinct shapes are revealed when the guts of 'regular' (Figure 3) and irregular (Figure 4) sea urchin specimens are compared. After the scanning process, a digital photograph was taken of every specimen selected for this study (Figures 3B and 4B).

MRI also revealed a striking difference regarding the architecture of the gastric caecum. Except for the two clypeasteroid subtaxa Laganina and Scutellina, this structure can be found in all irregular taxa (Figure 4C-E), located at the beginning of the stomach. Its presence correlates with the feeding habits of this group of sea urchins: most of these animals are partially or completely covered by sediment which they ingest to filter out organic material. Although the precise function of the gastric caecum is still a matter of discussion, it appears to have a major role in digestion (see, for example, [38, 39]). In specimens of comparable size, substantial differences in its size and shape were found (Figure 5). The highly reduced gastric caecum consisting of numerous smaller blindly ending sacs that can be observed in *Echinolampas depressa* Gray, 1851 (Figure 5B) appears to be a common characteristic which sets the Cassiduloida apart from other taxa (Figure 5A, C and D) of the Irregularia. This is emphasized by the presence of a similar, highly reduced gastric caecum in the cassiduloid species *Cassidulus caribearum* Lamarck, 1801 (data not shown; as described also by Gladfelter [40]) and *Rhyncholampas pacificus* Agassiz, 1863 (as shown in [41]). A comprehensive description and comparative analysis of sea urchin internal anatomy will be published elsewhere (Ziegler et al., publication in preparation).

Interactive viewing of selected species in three dimensions

To illustrate the opportunities offered by digitized reconstructions, Figure 6A-C shows different views of the 3D model of a member of the Cidaroida (or pencil urchins), *E. metularia*. This widely distributed Indo-Pacific species possesses a moderately thick calcite endoskeleton, large primary and short secondary spines. Its internal organization is characterized by the presence of five bushy Stewart's organs, five stalked gonads and a short intestine. The drawing presented in Figure 6D was made by Stewart [42] from a closely related species, *Cidaris cidaris* (= *Dorocidaris papillata*) (Linnaeus, 1758), where he depicts the newly discovered organs that were later named after him. A comparison of his drawing with the digital model in Figure 6C demonstrates that modern imaging protocols in combination with 3D capability may serve as a valuable substitute for laborious and subjective traditional dissection and visualization techniques. The interactive 3D model of *E. metularia* (accessible through a click onto Figure 6 in the 3D PDF version of this article: Additional file 7) permits, apart from standard operations such as zoom and drag, the selection of the numerous reconstructed structures. These elements can individually be made transparent for an unobstructed view on all organs they may occlude. Several pre-installed views can be selected as well. The creation of this model, including MRI data acquisition, took less than three full work days. All other models shown in Figures 3 and 4 are available for download and interactive viewing on The Echinoid Directory [43].

Discussion

Methodology

The initial phase of our study served to determine the proper imaging conditions using high-field MRI instruments. In order to achieve resolutions below $(100\ \mu m)^3$, we employed 7 T and 17.6 T small animal scanners. We were able to obtain resolutions ranging from $20 \times 18 \times 18\ \mu m^3$ to $(86\ \mu m)^3$ with specimens ranging from 5 mm to 3.4 cm in size. Therefore, in terms

of resolution, our data is comparable to results derived from manual dissection techniques in combination with stereomicroscopic observation. Susceptibility artefacts were primarily seen in the sediment-feeding irregular species, with the gut content being the source of these artefacts (see, for example, Additional file 6, *Echinocyamus pusillus* (Müller, 1776)). What sediment component is causing which type of artefact needs to be determined in mineralogical studies on freshly fixed specimens. The effects of the contrast agent made it possible to reduce the negative effects of these artefacts in some cases (Figure 1C and D), but a thorough investigation of the potential of selective and non-selective contrast agents [44] is essential for their future application in invertebrate morphology. Other major sources for artefacts were the tendency of spatangoid sea urchins to accumulate ferric iron phosphate in the connective tissues of their digestive tracts [45], and the digestive tract diverticula of scutelline sand dollars known to harbour magnetite [46]. However, the scanning of several specimens of a given species and the matching of their internal structures did in some cases help to overcome these problems.

One of the big advantages of MRI techniques is the extremely simple preparation of fixed specimens, being limited to the watering of the specimens (possibly in the presence of a contrast agent) and later putting them back into alcohol for storage. No detrimental effects of this procedure were noted during our studies. Apart from morphological studies on fixed specimens, MRI can also be used for *in vivo* analyses of invertebrates where, in contrast to μCT techniques that are based on X-rays, the living specimen is not harmed during the scanning process. Extensive morphological or developmental studies can thus be carried out, although anaesthetic substances may have to be applied to reduce movement artefacts during the long scanning times that are needed for high-resolution datasets and 3D reconstructions. Another very important advantage of non-invasive imaging techniques in general is the fact that the digitally acquired image stacks do not have to be manually aligned, as is the case with

digital pictures from histological or electronmicroscopical sections. Although these datasets can be used to generate highly resolved digitized 3D models of invertebrates as well (see, for example, [47, 48]), these analyses are extremely demanding in terms of specimen preparation, slice alignment and 3D reconstruction. In comparison, 3D reconstruction of MRI data is simple and highly accurate, but the segmentation process carried out for the present work is still subjective and our primary hypotheses regarding organ designation had to be evaluated against the information available from classical histological literature. However, user-friendly semi-automatic segmentation [49] as well as artefact and noise reduction [50] algorithms have recently become available, and may further simplify the task of 3D reconstruction. The highly demanding step of obtaining a completely automated segmentation and reconstruction of an entire animal would be very desirable and appears technically feasible, although it is not yet available for comparative morphological studies.

In spite of the many positive features, a number of negative aspects are currently inherent to morphological studies of small animals using MRI. The achievable resolution is comparatively low, and results derived from the reconstruction of internal structures are currently comparable only to manual dissection. Furthermore, an exact outline of how future experiments on other invertebrate taxa should be performed can only be partially derived from our study, since imaging results of fixed specimens depend on several factors, some of them still not fully understood (for example, susceptibility artefacts or changes to organ systems after fixation). Another drawback is the fact that only limited *in vitro* staining possibilities are available for MRI studies, although some substances (for example manganese) can be used for *in vivo* staining [28]. A further disadvantage of MRI is that specimens cannot currently be scanned in alcohol, since this would require spectroscopic imaging techniques resulting in much longer scanning times at the desirable resolutions below $(100\ \mu m)^3$. In addition, in the case of sea urchins, the 3D models generated (see, for example, Figure 6) are practically

useless for taxonomic purposes since sea urchin taxonomy is currently based entirely on hard parts. Resolutions well below (10 µm)^3 would be necessary to differentiate between the often miniscule characters of the sea urchin skeleton. However, using µCT, these datasets can potentially be gathered, as demonstrated by µCT scans of two sea urchin species, *Strongylocentrotus purpuratus* (Stimpson, 1857) [51] and *Encope michelini* Agassiz, 1841 [52], presented on the Digital Morphology Library (DigiMorph) website [53].

On the other hand, the access to MRI instruments does not appear to be a limiting factor. There are currently more than 1000 experimental small animal MRI instruments worldwide available for pre-clinical imaging. Since all high-field MRI scanners consist of super-conducting electromagnets, overnight and weekend scans as performed during this study would permit an optimal use of this resource.

Internal morphology of sea urchins

Despite the existence of extensive fossil, morphological and molecular datasets for this taxon, comparatively little systematic knowledge has been gathered on the internal anatomy of sea urchins. However, some authors have greatly extended our knowledge about the internal anatomy of selected, readily available sea urchin species such as *Cidaris cidaris* (Linnaeus, 1758) [54], *Arbacia punctulata* (Lamarck, 1816) [55], *Paracentrotus lividus* (Lamarck, 1816) [56, 57], *Echinus esculentus* Linnaeus, 1758 [58], *Sphaerechinus granularis* (Lamarck, 1816) [57, 59], *Spatangus purpureus* Müller, 1776 [57] or *Echinocardium cordatum* (Pennant, 1777) [38], not to mention the extensive works of Alexander E Agassiz, Hubert L Clark and Theodor J Mortensen. A common drawback of these studies is that a given author rarely dissected and analysed several species of a single taxon using one consistent method. Only in very few cases have histological sections, dissected specimens and so on, been preserved. The conclusions drawn from the original data can therefore not always be scrutinized.

Furthermore, the results were sometimes biased by prevailing morphological hypotheses. For example, recent histological studies [60] as well as our findings (Figure 3) indicate that the siphon, a canal bypassing the sea urchin stomach that was believed not to be found in the Diadematoida (see, for example, [61]), is indeed also present in this group of animals. Distinct features of internal sea urchin anatomy may extend to the sizes and shapes of digestive tract, gonads, ampullae and muscles. A thorough revision of internal structures in the light of recent technological advances will therefore almost certainly change our view of facts that, for years, were taken for granted. Scanning of sea urchins has so far been performed by us on more than 50 species (Ziegler et al., publication in preparation).

The feasibility of comparative morphological studies using MRI is further exemplified by our description of the highly variable shape and size of the gastric caecum found in the Irregularia (Figure 5). Our findings were correlated with the little information available on this structure; the combined data led us to assume that a reduced gastric caecum consisting of several smaller blindly ending sacs might constitute a soft tissue character common to all cassiduloid sea urchins. We therefore predict the presence of a similarly shaped structure in all species of the Echinolampadidae and Cassidulidae. Furthermore, it would be of interest to extend our analyses to members of the Apatopygidae and Neolampadidae, the other two extant families currently assigned to the Cassiduloida.

Although for some organs high intraspecific variability was detected, in particular in the case of organs with a development-dependent size and shape such as the gonads (Figure 1E and F), shape and localization of other organs (for example the digestive tract) did not differ significantly among specimens from a given echinoid species, allowing comparison on various taxic levels (Figures 3 and 4). However, intraspecific variability of internal organs can

also be observed in other taxa, for example gastropods (sinistral freak) or humans (situs inversus viscerum). Therefore, like larval and hard-part morphology, which are continuously updated [62, 63], morphological character sets derived from internal organs of sea urchins will serve as a useful systematic reference for future studies.

Non-invasive imaging techniques other than MRI

A number of imaging techniques allow for non-invasive morphological studies on whole specimens. However, only a few of them are able to achieve resolutions that permit detailed morphological analyses in small animals. Confocal laser scanning microscopy (CLSM), in combination with the autofluorescence of the crustacean or insect cuticula, can be used for 3D reconstructions of minuscule body parts or whole specimens [64-66]. The potential of this promising technique must not be underestimated, since the achievable resolutions permit taxonomic studies (based, for example, on chaetotaxy) as well as large-scale systematic morphological analyses. However, this procedure reveals internal structures only in rare cases and is limited to tiny objects.

In contrast to CLSM, µCT has already become a standard tool for detailed non-invasive studies of whole specimens, especially in studies on fossils [67]. It is based on the analysis of a sample by means of X-rays and can primarily be used to reveal hard tissue structures such as bone or calcite. Moreover, recent studies using more advanced techniques such as X-ray synchrotron microtomography and a new setup called very-high-resolution X-ray computed tomography have been employed to display soft tissues, *in vitro* as well as *in vivo* [6, 7, 68-70] (see also the review by Attwood [4]). Using µCT, isotropic resolutions of less than $(5\ \mu m)^3$ have been achieved on whole specimens, although, technically, resolutions in the nanometre scale are within reach. However, whether these new µCT methods can compete with the ability of MRI to display soft tissue at high resolutions still needs to be evaluated.

Our own preliminary studies to generate images of internal organs performed on several sea urchin museum specimens (*Echinoneus cyclostomus* Leske, 1778, *Psammechinus miliaris* (Müller, 1771), *Mespilia globulus* (Linnaeus, 1758), and *Moira atropos* (Lamarck, 1816)) using a desktop high-resolution µCT scanner (Skyscan 1172, Skyscan, Kontich, Belgium) at the Max-Planck-Institut für evolutionäre Anthropologie, Leipzig, Germany were not successful, although these µCT datasets can be used for the reconstruction of hard tissue structures. 3D models based on this data will be made available on the DigiMorph website [53].

These results demonstrate that complementary imaging techniques should ideally be used for comparative morphological analyses, an approach commonly referred to as multimodality.

Conclusions

Our results extend the use of MRI in invertebrate morphology to a systematic approach using museum specimens of a major marine invertebrate taxon, the sea urchins (Echinoidea). MRI can be employed for the rapid, non-destructive and unbiased acquisition of digital morphological data, and the creation of interactive 3D models accessible both within a publication and via the Web. The revelation of the internal anatomy of echinoid museum specimens at a resolution comparable with manual dissection extends our knowledge of soft tissue characters, permitting phylogenetic inferences.

Methods

Specimen preparation

All specimens analyzed belong to the taxon Echinoidea (sea urchins), one of the five subtaxa of the Echinodermata (marine spiny-skinned animals). The museum specimens were mostly fixed in formalin and all were conserved in alcohol, although in some cases the exact mode of

fixation could not be determined. For MR scanning, the museum specimens had to be lowered down to distilled water in a gradual alcohol series. Specimens that were obtained in living state were fixed in a 7% formaldehyde solution. For MR scanning, the freshly fixed specimens were kept in this solution. Results derived from specimens scanned in alcohol and formalin did not appear to differ significantly.

The specimens where placed either inside a custom-built Plexiglas chamber (Berlin), inside a 50 ml Falcon tube (Berlin), or inside NMR tubes with diameters between 5 and 20 mm (Würzburg), depending on the size of the specimen. On some specimens spines had to be dressed for tight fit inside the tubes. Magnevist (Bayer-Schering, Berlin, Germany), a gadolinium-based non-selective contrast agent, was added at a final concentration of 2 mM. This concentration had been employed successfully in preliminary studies (results not shown). The addition of the contrast agent to the museum specimens was discussed beforehand with the curators who judged the risk to the specimens' integrity as negligible. Prior to scanning, samples were degassed at 50 mbar. The freshly fixed specimens used in this study will be deposited as voucher material at the ZMB. Museum specimens will be stored in separate jars for potential later re-scanning. Table 2 lists detailed information on every museum and freshly fixed specimen used in this study.

Specimen scanning and photography

Berlin

Experiments were conducted at the Berlin NeuroImaging Center, Charité - Universitätsmedizin Berlin, Germany on a 7 T Pharmascan 70/16 AS rodent scanner with a ^{1}H-resonance frequency of 300 MHz (Bruker Biospin GmbH, Ettlingen, Germany). The system consisted of a 160 mm horizontal bore magnet, a shielded gradient set with an inner diameter of 90 mm, and a maximum gradient strength of 300 mT/m. A ^{1}H-radio frequency

linear transmit/receive birdcage resonator with an inner diameter of 38 mm was used for excitation and signal detection. The images described here were acquired at around 18°C. Data acquisition and image processing were carried out with Paravision 4.0.

Würzburg

Experiments were conducted at the Physikalisches Institut, Würzburg, Germany at 17.6 T with a ^{1}H-resonance frequency of 750 MHz on a Bruker Avance 750WB NMR spectrometer (Bruker Biospin GmbH, Rheinstetten, Germany). The system consisted of an 89 mm vertical bore magnet, a shielded gradient set with an inner diameter of 40 mm, and a maximum gradient strength of 1 T/m. A 20 mm linear birdcage resonator and a 5 mm linear bird cage resonator were used. The images described here were acquired at around 18°C. Image processing was carried out with Paravision 3.0.2 and involved zero filling to a $(512)^3$ matrix prior to Fourier transformation.

Scanning protocols employed a 3D gradient echo imaging sequence (FLASH) with parameters depending on the specimen size (see Additional file 2 for details). Almost all scans resulted in datasets with isotropic spatial resolution.

The photos of museum and freshly fixed specimens were made using a digital camera (Casio Exilim) with 7.2 Megapixels. The acquired images were processed using Adobe Photoshop CS2 9.0.

3D visualization

3D image reconstruction and visualization were performed by converting the generated MRI data (DICOM standard) into an 8-bit TIFF image sequence (ImageJ 1.38w) and by using 3D imaging software (amira 3.0.2, Mercury Computer Systems, Berlin, Germany). Segmentation

was carried out manually by using the brush tool in the amira Image Segmentation Editor. In some cases structures on every other slice were labelled, with subsequent ‘interpolation’ of structures on intervening slices, followed by a check and correction of segmentation results if necessary. Detection of borderlines and organ designations were performed based on literature, our own histological data and previously acquired MRI data [31, 32].

Operations were carried out on a desktop PC (CPU: Intel Core 2 Duo, 2.67 GHz; graphics card: NVIDIA GeForce 8600 GT; operating system: Windows XP; display: WACOM Cintiq 21UX Pen Display). Image processing was performed using Adobe Photoshop CS2 9.0 and Adobe Illustrator CS2 12.0.1. The movies were produced using the ImageJ 1.38w QuickTime export function. The individual 3D structures were saved as Wavefront files and imported into Adobe 3D Toolkit 8.1.0, where they were reassembled and transformed into an interactive 3D PDF. The 3D model was embedded into this publication using the Adobe 3D Toolkit according to the procedures described in [71].

List of abbreviations

3D, three-dimensional; BAH, Biologische Anstalt Helgoland, Germany; CAS, California Academy of Sciences, San Francisco, USA; CLSM, confocal laser scanning microscopy; µCT, micro-computed tomography; FOV, field of view; MRI, magnetic resonance imaging; NHM, Natural History Museum, London, UK; NMR, nuclear magnetic resonance; USNM, National Museum of Natural History, Washington DC, USA; ZMB, Systematische Zoologie am Museum für Naturkunde, Berlin, Germany.

Authors’ contributions

AZ designed and coordinated the experiments, carried out the dissections, prepared specimens and carried out scanning and 3D modelling. CF and SM prepared and scanned specimens. TB

supervised the experiments. All authors contributed to writing the manuscript and approved of the final version.

Acknowledgements

The authors thank Andrew Cabrinovic (NHM), Carsten Lüter (ZMB), David Pawson (USNM), and Rich Mooi (CAS) for the generous supply with sea urchin museum specimens. We sincerely appreciate the help with photography from Peter Adam (Institut für Biologie, Freie Universität Berlin, Germany). Andrew B Smith (NHM) is thanked for introducing AZ to sea urchin systematics and commenting on the manuscript. We are grateful to Heiko Temming (Max-Planck-Institut für evolutionäre Anthropologie, Leipzig, Germany) for carrying out the μCT scans. The authors appreciate the comments by Timothy Rowe (The University of Texas at Austin, Austin, USA) and two anonymous reviewers. We also thank Andreas Ziegler (Institut für Immungenetik, Freie Universität Berlin, Germany) for critical reading of the manuscript. The 17.6 T NMR spectrometer was funded by the DFG (Ha1232/13). AZ was supported through stipends from the State of Berlin (NaFöG), Germany, and the Friedrich-Naumann-Stiftung für die Freiheit, Potsdam, Germany

References

1. Hörnschemeyer T, Beutel RG, Pasop F: **Head structure of *Priacma serrata* Leconte (Coleoptera, Archostemata) inferred from x-ray tomography**. *J Morphol* 2002, **252:**298-314.

2. Wirkner CS, Richter S: **Improvement of microanatomical research by combining corrosion casts with microCT and 3D reconstruction, exemplified in the circulatory organs of the woodlouse.** *Microsc Res Tech* 2004, **64:**250-254.

3. Burrow CJ, Jones AS, Young G: **X-ray microtomography of 410 million-year-old optic capsules from placoderm fishes.** *Micron* 2005, **36:**551-557.

4. Attwood D: **Nanotomography comes of age.** *Nature* 2006, **442:**642-643.

5. Betz O, Wegst U, Weide D, Heethoff M, Helfen L, Lee WK, Cloetens P: **Imaging applications of synchrotron X-ray phase-contrast microtomography in biological morphology and biomaterials science. I. General aspects of the technique and its advantages in the analysis of millimetre-sized arthropod structure.** *J Microsc* 2007, **227:**51-71.

6. Dierick M, Cnudde V, Masschaele B, Vlassenbroeck J, Van Hoorebeke L, Jacobs P: **Micro-CT of fossils preserved in amber.** *Nucl Instrum Meth Phys Res A* 2007, **580:**641-643.

7. Socha JJ, Westneat MW, Harrison JF, Waters JS, Lee WK: **Real-time phase-contrast x-ray imaging: a new technique for the study of animal form and function.** *BMC Biol* 2007, **5:**6.

8. Wirkner CS, Richter S: **The circulatory system in Mysidacea - implications for the phylogenetic position of Lophogastrida and Mysida (Malacostraca, Crustacea).** *J Morphol* 2007, **268:**311-328.

9. Jones G, Christal M: **The future of virtual museums: on-line, immersive, 3D environments**. Created Realities Group, 2002 [http://tinyurl.com/4vt97g]

10. Rowe J: **Developing a 3D digital library for spatial data: issues identified and description of prototype.** *RLG DigiNews* 2002, **6:**5.

11. Budd GE, Olsson L: **A renaissance for evolutionary morphology**. *Acta Zool* 2007, **88:**1.

12. Brouwer M, Engel DW, Bonaventura J, Johnson GA: **In vivo magnetic resonance imaging of the blue crab, *Callinectes sapidus*: effect of cadmium accumulation in tissues on protein relaxation properties.** *J Exp Zool* 1992, **263:**32-40.

13. Bock C, Frederich M, Wittig RM, Pörtner HO: **Simultaneous observations of haemolymph flow and ventilation in marine spider crabs at different temperatures: a flow-weighted MRI study.** *Magn Reson Imaging* 2001, **19:**1113-1124.

14. Gozansky EK, Ezell EL, Budelmann BU, Quast MJ: **Magnetic resonance histology: in situ single cell imaging of receptor cells in an invertebrate (*Lolliguncula brevis*, Cephalopoda) sense organ.** *Magn Reson Imaging* 2003, **21:**1019-1022.

15. Herberholz J, Mims CJ, Zhang X, Hu X, Edwards DH: **Anatomy of a live invertebrate revealed by manganese-enhanced magnetic resonance imaging.** *J Exp Biol* 2004, **207:**4543-4550.

16. Brinkley CK, Kolodny NH, Kohler SJ, Sandeman DC, Beltz BS: **Magnetic resonance imaging at 9.4 T as a tool for studying neural anatomy in non-vertebrates.** *J Neurosci Meth* 2005, **146:**124-132.

17. Davenel A, Quellec S, Pouvreau S: **Noninvasive characterization of gonad maturation and determination of the sex of Pacific oysters by MRI.** *Magn Reson Imaging* 2006, **24:**1103-1110.

18. Pouvreau S, Rambeau M, Cochard JC, Robert R: **Investigation of marine bivalve morphology by in vivo MR imaging: first anatomical results of a promising technique.** *Aquaculture* 2006, **259:**415-423.

19. Pohlmann A, Möller M, Decker H, Schreiber WG: **MRI of tarantulas: morphological and perfusion imaging.** *Magn Reson Imaging* 2007, **25:**129-135.

20. Müller WEG, Kaluzhnaya OV, Belikov SI, Rothenberger M, Schröder HC, Reiber A, Kaandorp JA, Manz B, Mietchen D, Volke F: **Magnetic resonance imaging of the siliceous skeleton of the demosponge *Lubomirskia baicalensis*.** *J Struct Biol* 2006, **153:**31-41.

21. Goodman BA, Gordon SC, Chudek JA, Hunter G, Woodford JAT: **Nuclear magnetic resonance microscopy as a non-invasive tool to study the development of lepidopteran pupae.** *J Insect Physiol* 1995, **41:**419-424.

22. Mapelli M, Greco F, Gussoni M, Consonni R, Zetta L: **Application of NMR microscopy to the morphological study of the silkworm, *Bombyx mori*, during its metamorphosis.** *Magn Reson Imaging* 1997, **15:**693-700.

23. Jasanoff A, Sun PZ: ***In vivo* magnetic resonance microscopy of brain structure in unanesthetized flies**. *J Magn Reson Ser A* 2002, **158:**79-85.

24. Wecker S, Hörnschemeyer T, Hoehn M: **Investigation of insect morphology by MRI: assessment of spatial and temporal resolution.** *Magn Reson Imaging* 2002, **20:**105-111.

25. Haddad D, Schaupp F, Brandt R, Manz G, Menzel R, Haase A: **NMR imaging of the honeybee brain.** *J Insect Sci* 2004, **4:**7.

26. Huetteroth W, Schachtner J: **Standard three-dimensional glomeruli of the *Manduca sexta* antennal lobe: a tool to study both developmental and adult neuronal plasticity.** *Cell Tissue Res* 2005, **319:**513-524.

27. Michaelis T, Watanabe T, Natt O, Boretius S, Frahm J, Utz S, Schachtner J: ***In vivo* 3D MRI of insect brain: cerebral development during metamorphosis of *Manduca sexta*.** *Neuroimage* 2005, **24:**596-602.

28. Watanabe T, Schachtner J, Krizan M, Boretius S, Frahm J, Michaelis T: **Manganese-enhanced 3D MRI of established and disrupted synaptic activity in the developing insect brain in vivo.** *J Neurosci Meth* 2006, **158:**50-55.

29. Hörnschemeyer T, Goebbels J, Weidemann G, Faber C, Haase A: **The head morphology of *Ascioplaga mimeta* (Coleoptera: Archostemata) and the phylogeny of Archostemata.** *Eur J Entomol* 2006, **103:**409-423.

30. Hart AG, Bowtell RW, Köckenberger W, Wenseleers T, Ratnieks FLW: **Magnetic resonance imaging in entomology: a critical review.** *J Insect Sci* 2003, **3:**5.

31. Ziegler A, Angenstein F: **Analyse von Seeigeln (Echinoidea) mit Hilfe der bildgebenden Magnetresonanztomographie.** *Mikrokosmos* 2007, **96:**49-54.

32. Ziegler A, Mueller S, Bartolomaeus T: **Sea urchin (Echinoidea) anatomy revealed by magnetic resonance imaging and 3D visualization.** In *Proceedings of the 12th International Echinoderm Conference.* Edited by Harris LG. Rotterdam: AA Balkema; in press.

33. Dunn CW, Hejnol A, Matus DQ, Pang K, Browne WE, Smith SA, Seaver E, Rouse GW, Obst M, Edgecombe GD, Sørensen MV, Haddock SHD, Schmidt-Rhaesa A, Okusu A, Kristensen RM, Wheeler WC, Martindale MQ, Giribet G: **Broad phylogenomic sampling improves resolution of the animal tree of life.** *Nature* 2008, **452:** 745-749**.**

34. Littlewood DTJ, Smith AB: **A combined morphological and molecular phylogeny for sea urchins (Echinoidea: Echinodermata).** *Philos Trans R Soc Lond B Biol Sci* 1995, **347:**213-234.

35. Smith AB, Pisani D, Mackenzie-Dodds JA, Stockley B, Webster BL, Littlewood DTJ: **Testing the molecular clock: molecular and paleontological estimates of divergence times in the Echinoidea (Echinodermata).** *Mol Biol Evol* 2006, **23:**1832-1851.

36. Sea Urchin Genome Sequencing Consortium: **The genome of the sea urchin *Strongylocentrotus purpuratus*.** *Science* 2006, **314:**941-952.

37. Ruppert EE, Fox RS, Barnes RD: *Invertebrate Zoology - A Functional Evolutionary Approach*. Belmont: Brooks/Cole-Thomson Learning; 2004.

38. DeRidder C, Jangoux M: **The digestive tract of the spatangoid echinoid *Echinocardium cordatum* (Echinodermata): morphofunctional study.** *Acta Zool* 1993, **74:**337-351.

39. Thorsen MS: **Abundance and biomass of the gut-living microorganisms (bacteria, protozoa and fungi) in the irregular sea urchin *Echinocardium cordatum* (Spatangoida: Echinodermata).** *Mar Biol* 1999, **133:**335-360.

40. Gladfelter WB: **General ecology of the cassiduloid urchin *Cassidulus caribbearum*.** *Mar Biol* 1978, **47:**149-160.

41. Agassiz A: **Revision of the Echini.** In *Illustrated Catalogue of the Museum of Comparative Zoology at Harvard College, No. VII.* Cambridge: Cambridge University Press; 1872-1874:1-761.

42. Stewart C: **On certain organs of the Cidaridae.** *Trans Linn Soc - Lond* 1877, **1:**569-572.

43. **The Echinoid Directory** [http://tinyurl.com/33bn38]

44. Shapiro MG, Atanasijevic T, Faas H, Westmeyer GG, Jasanoff A: **Dynamic imaging with MRI contrast agents: quantitative considerations.** *Magn Reson Imaging* 2006, **24:**449-462.

45. Buchanan JB, Brown BE, Coombs TL, Pirie BJS, Allen JA: **The accumulation of ferric iron in the guts of some spatangoid echinoderms.** *J Mar Biol Assoc* 1980, **60:**631-640.

46. Seilacher A: **Constructional morphology of sand dollars.** *Paleobiology* 1979, **5:**191-221.

47. Dacosta S, Cunha CM, Simone LRL, Schrödl M: **Computer-based 3-dimensional reconstruction of major organ systems of a new aeolid nudibranch subspecies, *Flabellina engeli licianae* from Brazil (Gastropoda: Opisthobranchia).** *J Mollus Stud* 2007, **73:**339-353.

48. Ruthensteiner B, Lodde E, Schopf S: **Genital system development of *Williamia radiata* (Gastropoda, Siphonariidae).** *Zoomorphology* 2007, **126:**17-29.

49. Yushkevich PA, Piven J, Hazlett HC, Smith RG, Ho S, Gee JC, Gerig G: **User-guided 3D active contour segmentation of anatomical structures: significantly improved efficiency and reliability.** *Neuroimage* 2006, **31:**1116-1128.

50. Vlassenbroeck J, Dierick M, Masschaele B, Cnudde V, Van Hoorebeke L, Jacobs P: **Software tools for quantification of x-ray microtomography at the UGCT.** *Nucl Instrum Meth Phys Res A* 2007, **580:**442-445.

51. **The Digital Morphology Library - *Strongylocentrotus purpuratus*** [http://tinyurl.com/5x9apf]

52. **The Digital Morphology Library - *Encope michelini*** [http://tinyurl.com/4tfgbl]

53. **The Digital Morphology Library** [http://www.digimorph.org]

54. Prouho H: **Recherches sur le *Dorocidaris papillata* et quelques autres échinides de la Méditeranée.** *Arch Zool Exp Gén* 1887, **5:**213-380.

55. Coe WR: **Echinoderms of Connecticut.** *State Geological and Natural History Survey Bulletin No. 19.* State of Connecticut; 1912.

56. Valentin G: **Anatomie du genre *Echinus*.** In *Monographies d'Echinodermes.* Edited by Agassiz L. Neuchatel; 1841:1-126.

57. Koehler R: **Recherches sur les échinides des côtes de Provence.** *Ann Mus Hist Natur Marseilles Zool* 1883, **1:**1-139.

58. Chadwick HC: ***Echinus*.** In *L.M.B.C. Memoirs.* Edited by Herdman WA. Liverpool: Liverpool Marine Biology Committee; 1900.

59. Strenger A: ***Sphaerechinus granularis* (Violetter Seeigel), Anleitung zur makroskopischen und mikroskopischen Untersuchung.** In *Großes Zoologisches Praktikum*, *Band 18.* Edited by Siewing R. Stuttgart: Gustav Fischer; 1973**:**1-68.

60. Campos LS, Moura RB: **Macrostructure and evolution of the echinoid digestive system.** *Zoomorphology* 2008, **127:**135-141.

61. Holland ND, Ghiselin MT: **A comparative study of gut mucous cells in thirty-seven species of the class Echinoidea (Echinodermata).** *Biol Bull* 1970, **138:**286-305.

62. Mooi R: **Paedomorphosis, Aristotle's lantern, and the origin of the sand dollars (Echinodermata: Clypeasteroida).** *Paleobiology* 1990, **16:**25-48.

63. Smith AB, Stockley B: **Fasciole pathways in spatangoid echinoids: a new source of phylogenetically informative characters.** *Zool J Linn Soc* 2005, **144:**15-35.

64. Klaus AV, Kulasekera VL, Schawaroch V: **Three-dimensional visualization of insect morphology using confocal laser scanning microscopy.** *J Microsc* 2003, **212:**107-121.

65. Klaus AV, Schawaroch V: **Novel methodology utilizing confocal laser scanning microscopy for systematic analysis in athropods (Insecta).** *Integr Comp Biol* 2006, **46:**207-214.

66. Michels J: **Confocal laser scanning microscopy: using cuticular autofluorescence for high resolution morphological imaging in small crustaceans.** *J Microsc* 2007, **227:**1-7.

67. Sutton MD: **Tomographic techniques fort the study of exceptionally preserved fossils.** *Proc R Soc Lond B Biol Sci* 2008, **275:**1587-1593.

68. Nickel M, Donath T, Schweikert M, Beckmann F: **Functional morphology of *Tethya* species (Porifera): 1. Quantitative 3D-analysis of *Tethya wilhelma* by synchrotron radiation based x-ray microtomography.** *Zoomorphology* 2006, **125:**209-223.

69. Tafforeau P, Boistel R, Boller E, Bravn A, Brunet M, Chaimanee Y, Cloetens P, Feist M, Hoszowska J, Jaeger JJ, Kay RF, Lazzari V, Marivaux L, Nel A, Nemoz C, Thibault X, Vignaud P, Zabler S: **Applications of x-ray synchrotron microtomography for non-destructive 3D studies of paleontological specimens.** *Appl Phys A: Mater* 2006, **83:**195-202.

70. Penney D, Dierick M, Cnudde V, Masschaele B, Vlassenbroeck J, Van Hoorebeke L, Jacobs P: **First fossil Micropholcommatidae (Araneae), imaged in Eocene Paris amber using x-ray computed tomography.** *Zootaxa* 2007, **1623:**47-53.

71. Ruthensteiner B, Hess M: **Embedding 3D models of biological specimens in PDF publications.** *Microsc Res Tech*, in press.

Figure legends

Figure 1 - Establishment of imaging conditions using specimens of *Psammechinus miliaris*

(A), (B) Comparison of two freshly fixed specimens. Magnetic resonance imaging (MRI) sections at the height of Aristotle's lantern and digestive tract. Resolution: $(81\ \mu m)^3$, no contrast agent added. The two specimens show a high degree of similarity in their overall internal architecture. Arrows indicate paramagnetic gut content. (C), (D) Effects of a contrast agent on image quality. MRI sections at the height of perignathic girdle and lower stomach. Resolution: $(81\ \mu m)^3$. This freshly fixed specimen was scanned (C) before and (D) after the application of a contrast agent, Magnevist. Arrows indicate susceptibility artefacts.

(E), (F) Comparison of a freshly fixed and a museum specimen. MRI sections at the height of gonads and upper oesophagus. Resolution: $(81\ \mu m)^3$. The 135-year-old museum specimen (F) gives imaging results comparable to the freshly fixed specimen (E). Both specimens were scanned with contrast agent added. Orientation: ambulacrum II facing upwards. Scale bar: 0.5 cm. ac, axial complex; al, Aristotle's lantern; am, ampulla; es, oesophagus; go, gonad; im, interpyramidal muscle; in, intestine; is, inner marginal sinus; lm, lantern muscle; os, outer marginal sinus; pg, perignathic girdle; re, rectum; si, siphon; st, stomach; to, tooth.

Figure 2 - Selected horizontal magnetic resonance imaging sections of different sea urchin species taken from Additional files 3-6

(A) *Eucidaris metularia*. Resolution: $(81\ \mu m)^3$. Aristotle's lantern, gonads, Stewart's organs, and stomach can be seen. (B) *Psammechinus miliaris*. Resolution: $(44\ \mu m)^3$. Aristotle's lantern, lantern muscles, stomach, and ampullae are visible. (C) *Echinoneus cyclostomus*. Resolution: $(86\ \mu m)^3$. Digestive tract with oesophagus, gastric caecum, stomach, and rectum

are shown. (D) *Echinocyamus pusillus*. Resolution: 20 × 18 × 18 μm^3. Aristotle's lantern, gonads, stomach, and rectum are represented. Scale bar: (A)-(C) 0.5 cm; (D): 1 mm. al, Aristotle's lantern; am, ampulla; es, oesophagus; gc, gastric caecum; lm, lantern muscle; re, rectum; so, Stewarts' organ; st, stomach.

Figure 3 - Overview chart showing analyzed specimens of 'regular' sea urchins and corresponding 3D reconstructions of selected internal organs

(A) Information on species name, geographic distribution, and systematics. (B) Photograph of scanned specimen, aboral view. (C)-(E) 3D models of reconstructed selected internal organs, stepwise turned by 90°: (C) aboral view (interambulacrum 5 facing upwards); (D) lateral view (interambulacrum 5 at back); (E) oral view (interambulacrum 5 facing downwards). The buccal sacs of *Caenopedina mirabilis* as well as the siphon of *Stomopneustes variolaris* could not be seen on the magnetic resonance imaging sections. Scale bar: 1 cm. The colour legend specifies organ designation.

Figure 4 - Overview chart showing analyzed specimens of irregular sea urchins and corresponding 3D reconstructions of selected internal organs

(A) Information on species name, geographic distribution, and systematics. (B) Photograph of scanned specimen, aboral view. (C)-(E) 3D models of reconstructed selected internal organs, stepwise turned by 90°: (C) aboral view (ambulacrum III facing to the right); (D) lateral view (ambulacrum III facing to the right); (E) oral view (ambulacrum III facing to the right). Scale bar: 1 cm, except for *Echinocyamus pusillus*: 1 mm. The colour legend specifies organ designation.

Figure 5 - 3D reconstructions of the gastric caecum of selected irregular sea urchin species

The gastric caecum is a translucent body free of sediment and probably constitutes one of the main sites of digestion [38, 39]. The upper diagrams show an aboral view, the lower diagrams a lateral view with the anterior side (ambulacrum III) oriented towards the right-hand side. Arrows indicate the position of the junction of the gastric caecum with the stomach. (A) *Echinoneus cyclostomus,* Echinoneoida. (B) *Echinolampas depressa*, Cassiduloida. Species of this sea urchin taxon presumably all possess a highly reduced gastric caecum consisting of numerous small blindly ending sacs. (C) *Pourtalesia wandeli*, Holasteroida. (D) *Abatus cavernosus*, Spatangoida. Scale bar: 0.5 cm.

Figure 6 - Comparison of a digital 3D model with a traditional anatomical sketch

(A)-(C) *Eucidaris metularia*. Selected views taken from the interactive 3D model:
(A) external view; (B) external view with transparent test, internal organs visible; (C) external view with transparent test and all internal organs removed except for Stewart's organs and Aristotle's lantern. (D) *Cidaris cidaris* (= *Dorocidaris papillata*). Image taken from [42] and modified. Stewart's organs constitute extensions of the peripharyngeal coelom. Scale bar: 1 cm. The colour legend specifies organ designation. The interactive 3D mode can be accessed by clicking onto Figure 6 in the 3D PDF version of this article: Additional file 7 (Adobe Reader Version 7.1 or higher required).

Additional files

Additional file 1

Table1.pdf

PDF file

Table 1 – Scanning parameters for specimens used in this study.

Additional file 2

Table2.pdf

PDF file

Table 2 – List of specimens used in this study.

Additional file 3

Video1_Eucidaris_metularia.mov

QuickTime video file

Video 1 - MRI dataset of *Eucidaris metularia* (Lamarck, 1816). Resolution: $(81\ \mu m)^3$.

Additional file 4

Video2_Psammechinus_miliaris.mov

QuickTime video file

Video 2 - MRI dataset of *Psammechinus miliaris* (Müller, 1771). Resolution: $(44\ \mu m)^3$.

Additional file 5

Video3_Echinoneus_cyclostomus.mov

QuickTime video file

Video 3 - MRI dataset of *Echinoneus cyclostomus* Leske, 1778. Resolution: $(86\ \mu m)^3$.

Additional file 6

Video4_Echinocyamus_pusillus.mov

QuickTime video file

Video 4 - MRI dataset of *Echinocyamus pusillus* (Müller, 1776). Resolution: $20 \times 18 \times 18\ \mu m^3$.

Additional file 7

Ziegler_et_al_article_3D.pdf

PDF file with embedded 3D model

3D model – 3D PDF version of the entire article. By clicking anywhere onto Figure 6 the 3D model of *Eucidaris metularia* (Lamarck, 1816) can be interactively accessed. The 3D model is based on the virtual MRI sections seen in Video 1. In order to view the 3D model please install (or upgrade to) Adobe Reader Version 7.1 or higher.

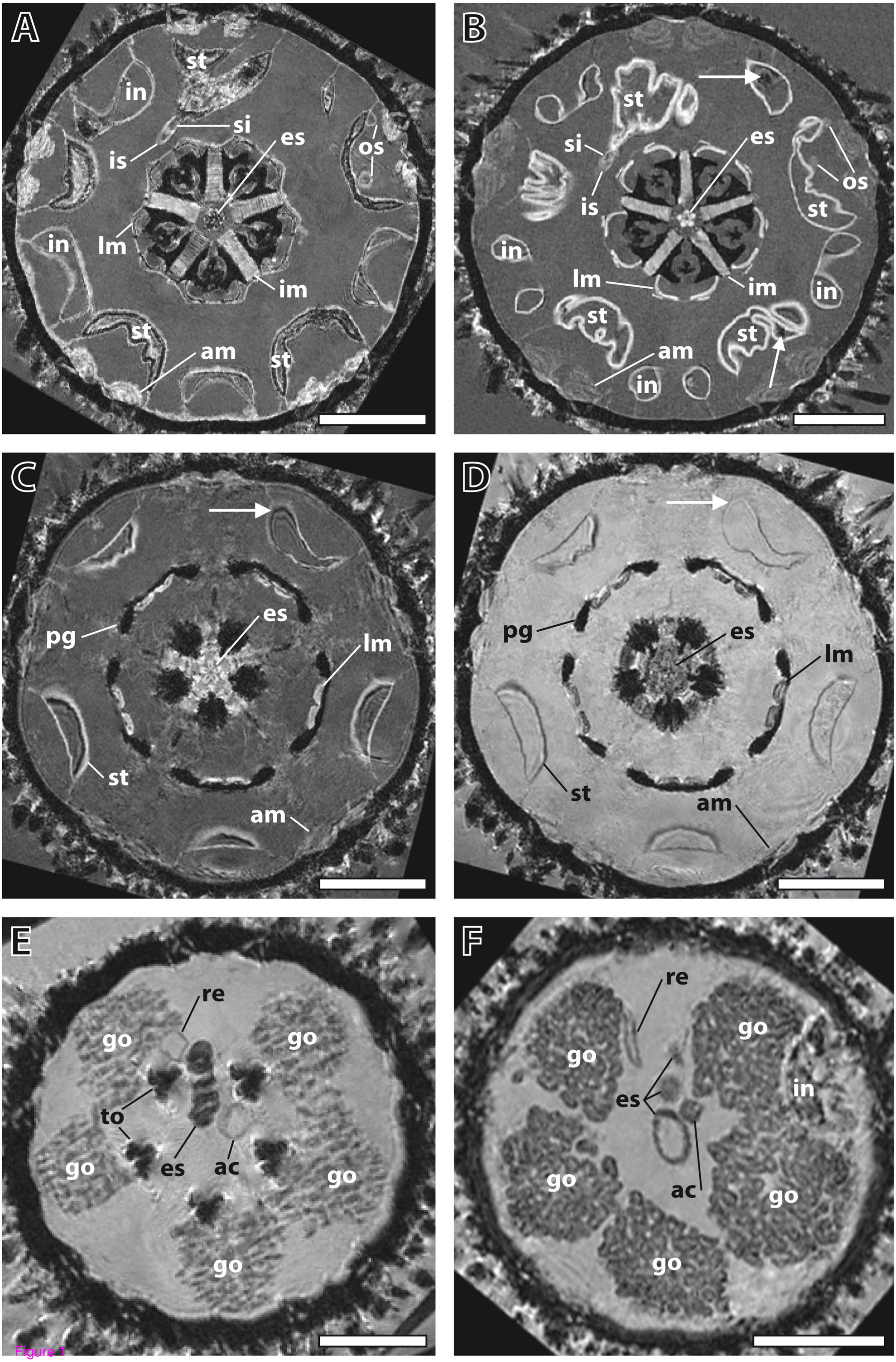
A
st
in
si
is
es
os
in
lm
im
st
am
st
B
st
es
si
is
os
st
in
lm
im
in
st
st
am
in
C
es
pg
lm
st
am
D
pg
es
lm
st
am
E
re
go
go
to
go
es
ac
go
go
F
re
go
go
in
es
go
ac
go
go
Figure 1

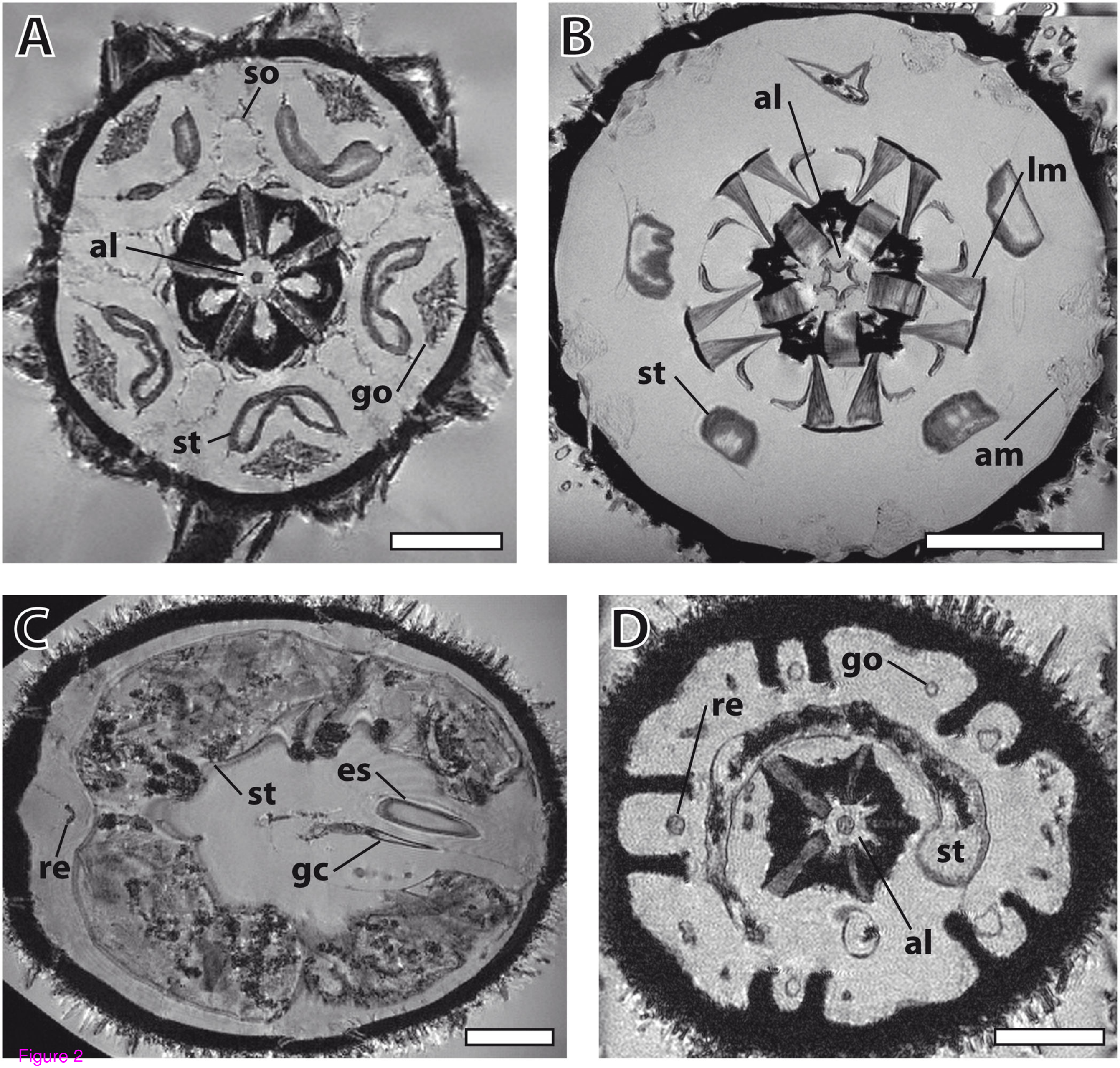

Figure 2

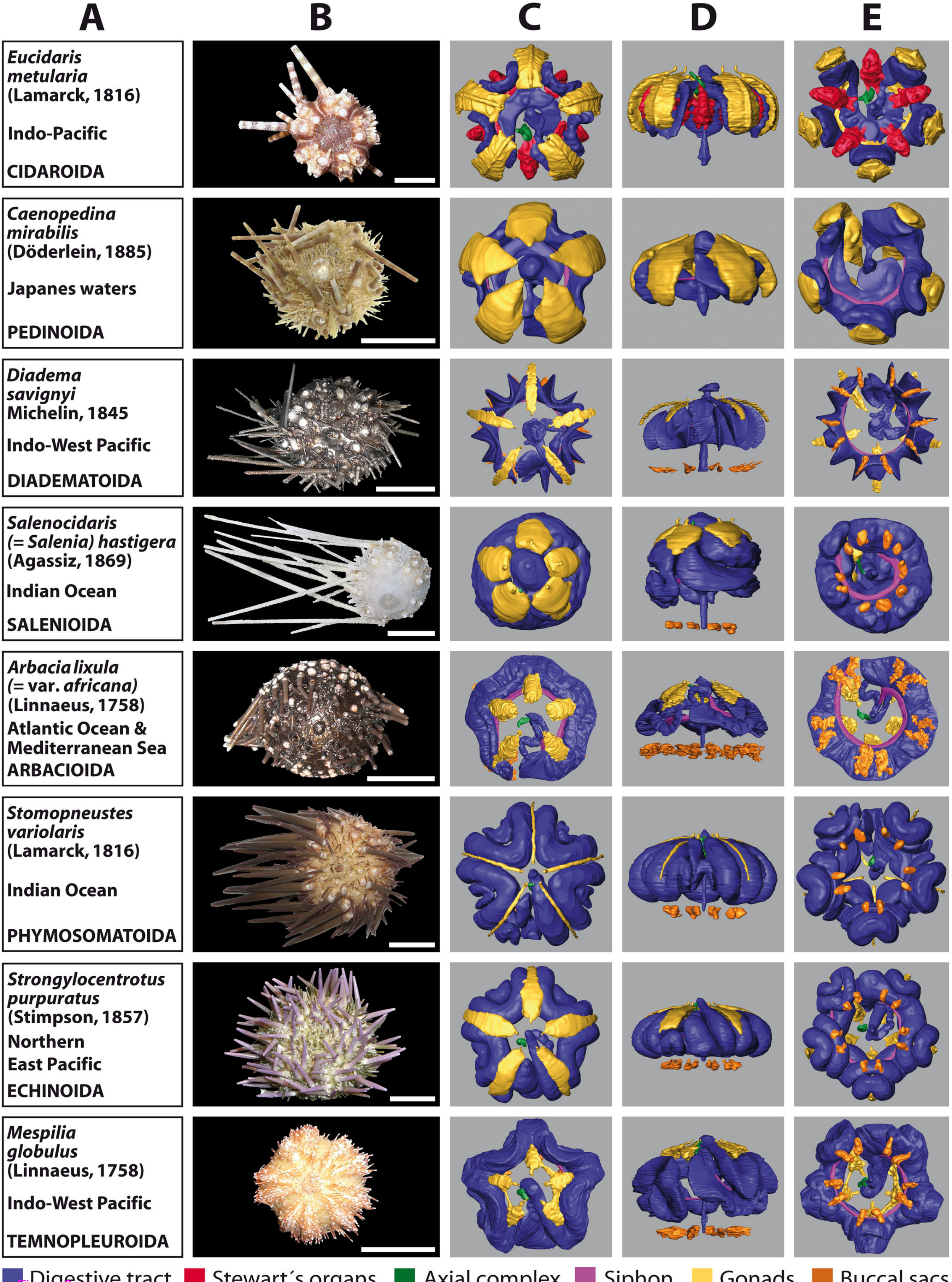
A
B
C
D
E
Eucidaris metularia (Lamarck, 1816)
Indo-Pacific
CIDAROIDA
Caenopedina mirabilis (Döderlein, 1885)
Japanes waters
PEDINOIDA
Diadema savignyi Michelin, 1845
Indo-West Pacific
DIADEMATOIDA
Salenocidaris (= Salenia) hastigera (Agassiz, 1869)
Indian Ocean
SALENIOIDA
Arbacia lixula (= var. africana) (Linnaeus, 1758)
Atlantic Ocean & Mediterranean Sea
ARBACIOIDA
Stomopneustes variolaris (Lamarck, 1816)
Indian Ocean
PHYMOSOMATOIDA
Strongylocentrotus purpuratus (Stimpson, 1857)
Northern East Pacific
ECHINOIDA
Mespilia globulus (Linnaeus, 1758)
Indo-West Pacific
TEMNOPLEUROIDA
Digestive tract
Stewart´s organs
Axial complex
Siphon
Gonads
Buccal sacs

Figure 3

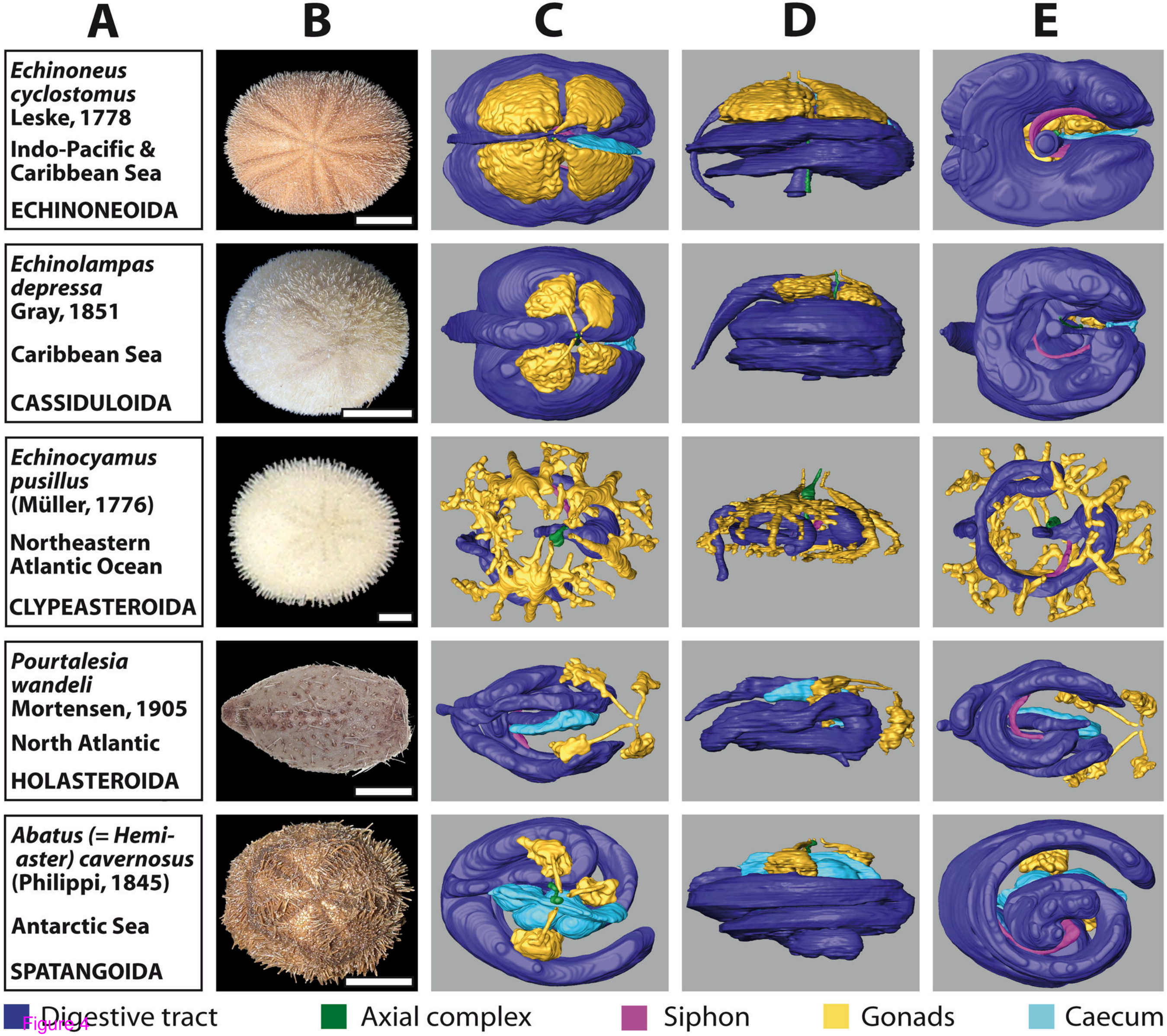
A
B
C
D
E
Echinoneus cyclostomus Leske, 1778
Indo-Pacific & Caribbean Sea
ECHINONEOIDA
Echinolampas depressa Gray, 1851
Caribbean Sea
CASSIDULOIDA
Echinocyamus pusillus (Müller, 1776)
Northeastern Atlantic Ocean
CLYPEASTEROIDA
Pourtalesia wandeli Mortensen, 1905
North Atlantic
HOLASTEROIDA
Abatus (= Hemi-aster) cavernosus (Philippi, 1845)
Antarctic Sea
SPATANGOIDA
Digestive tract
Axial complex
Siphon
Gonads
Caecum

Figure 4

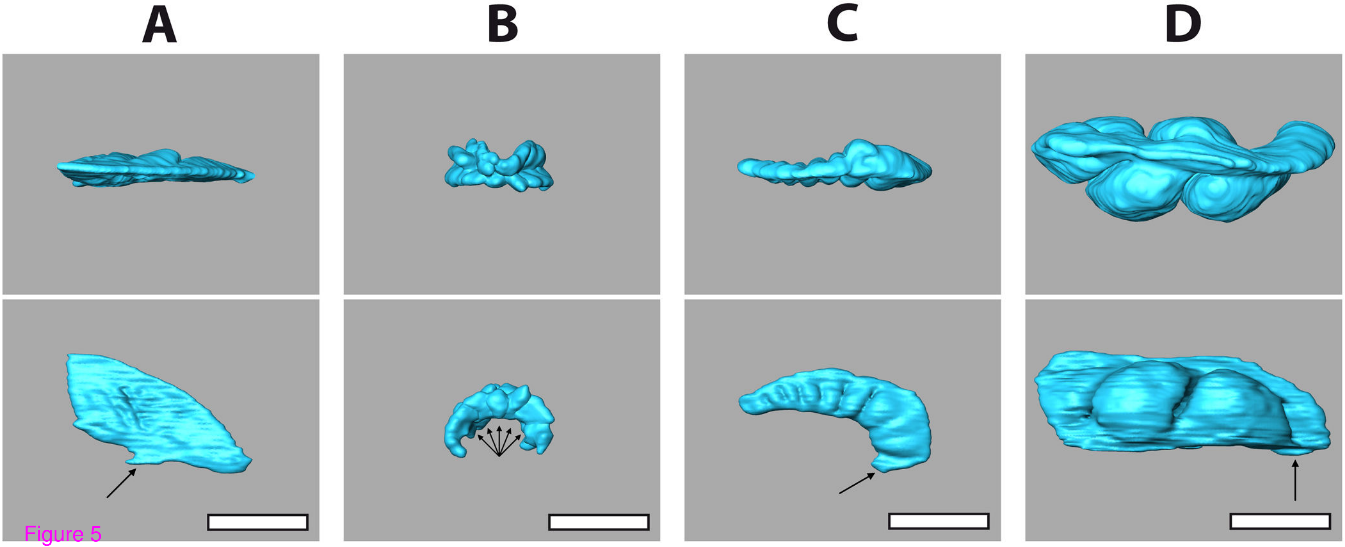

Figure 5

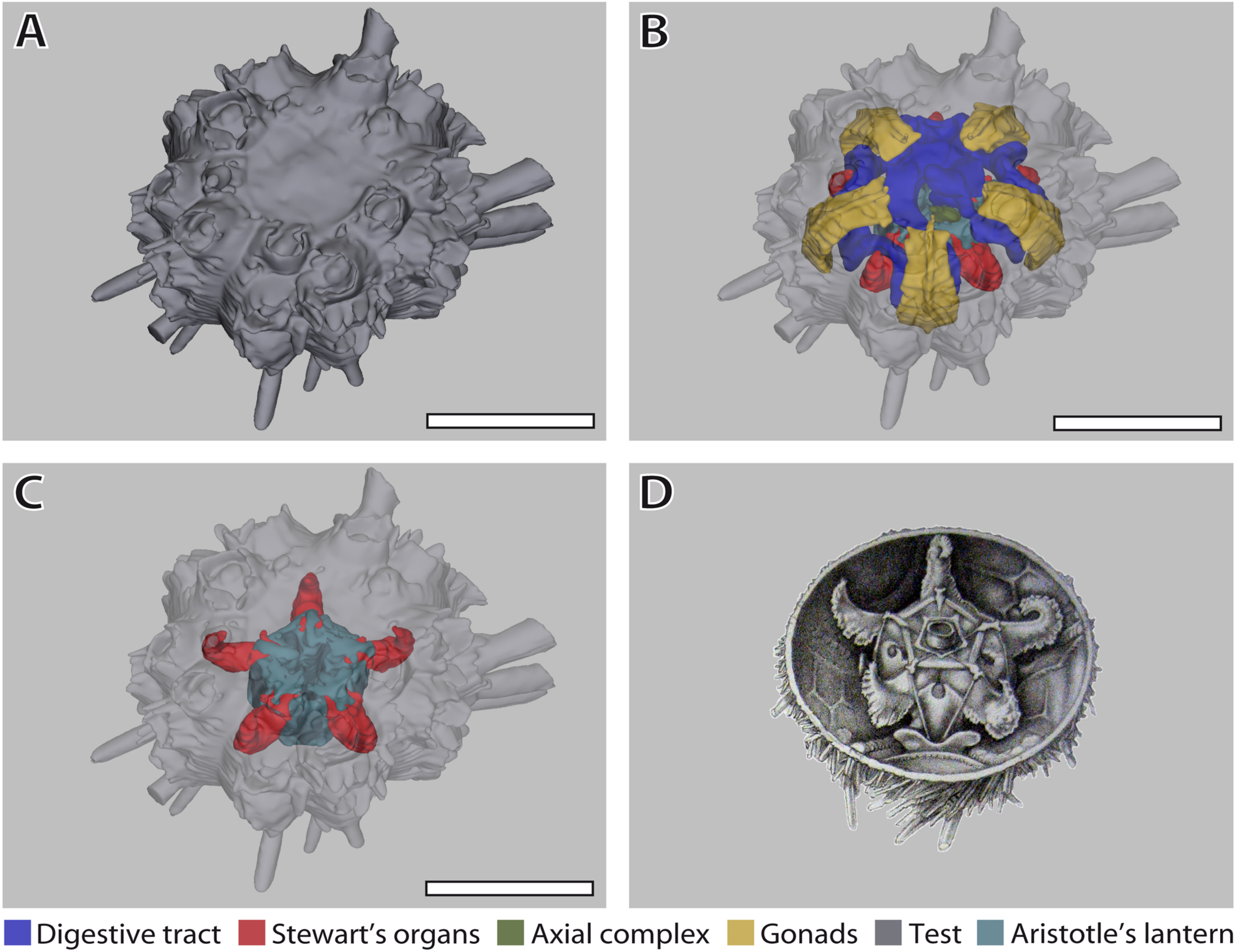
A
B
C
D
Digestive tract
Stewart's organs
Axial complex
Gonads
Test
Aristotle's lantern

Additional files provided with this submission:

Additional file 1: zeigler additional file 1 08-07.doc, 41K
http://www.biomedcentral.com/imedia/1336314483213059/supp1.doc
Additional file 2: zeigler additional file 2 08-07.doc, 34K
http://www.biomedcentral.com/imedia/8092494242110821/supp2.doc
Additional file 3: zeigler additional file 3.mov, 1070K
http://www.biomedcentral.com/imedia/1179741372110821/supp3.mov
Additional file 4: zeigler additional file 4.mov, 2272K
http://www.biomedcentral.com/imedia/8811235232110813/supp4.mov
Additional file 5: zeigler additional file 5.mov, 1359K
http://www.biomedcentral.com/imedia/3450129152110821/supp5.mov
Additional file 6: zeigler additional file 6.mov, 1532K
http://www.biomedcentral.com/imedia/1355957692211082/supp6.mov
